# A novel quinoline with airway relaxant effects and anti-inflammatory properties

**DOI:** 10.1186/s12931-024-02780-8

**Published:** 2024-03-30

**Authors:** Jesper Bergwik, Jielu Liu, Médea Padra, Ravi K. V. Bhongir, Lloyd Tanner, Yujiao Xiang, Mia Lundblad, Arne Egesten, Mikael Adner

**Affiliations:** 1 Department of Clinical Sciences Lund, Respiratory Medicine, Allergology, & Palliative Medicine, Lund University and Skåne University Hospital, Lund, Sweden; 2 Experimental Asthma and Allergy Research Unit, Institute of Environmental Medicine (IMM), Karolinska Institutet, Biomedicum, Solnavägen 9, 171 65 Stockholm, Sweden; 3 Arcede Pharma AB, Medicon Village, Lund, Sweden

**Keywords:** Airway hyperreactivity, Asthma, COPD, Lipopolysaccharide, Mitochondria, Ovalbumin

## Abstract

**Background:**

In chronic pulmonary diseases characterized by inflammation and airway obstruction, such as asthma and COPD, there are unmet needs for improved treatment. Quinolines is a group of small heterocyclic compounds that have a broad range of pharmacological properties. Here, we investigated the airway relaxant and anti-inflammatory properties of a novel quinoline (RCD405).

**Methods:**

The airway relaxant effect of RCD405 was examined in isolated airways from humans, dogs, rats and mice. Murine models of ovalbumin (OVA)-induced allergic asthma and LPS-induced airway inflammation were used to study the effects in vivo. RCD405 (10 mg/kg) or, for comparisons in selected studies, budesonide (3 mg/kg), were administered intratracheally 1 h prior to each challenge. Airway responsiveness was determined using methacholine provocation. Immune cell recruitment to bronchi was measured using flow cytometry and histological analyses were applied to investigate cell influx and goblet cell hyperplasia of the airways. Furthermore, production of cytokines and chemokines was measured using a multiplex immunoassay. The expression levels of asthma-related genes in murine lung tissue were determined by PCR. The involvement of NF-κB and metabolic activity was measured in the human monocytic cell line THP-1.

**Results:**

RCD405 demonstrated a relaxant effect on carbachol precontracted airways in all four species investigated (potency ranking: human = rat > dog = mouse). The OVA-specific IgE and airway hyperresponsiveness (AHR) were significantly reduced by intratracheal treatment with RCD405, while no significant changes were observed for budesonide. In addition, administration of RCD405 to mice significantly decreased the expression of proinflammatory cytokines and chemokines as well as recruitment of immune cells to the lungs in both OVA- and LPS-induced airway inflammation, with a similar effect as for budesonide (in the OVA-model). However, the effect on gene expression of *Il-4, IL-5* and *Il-13* was more pronounced for RCD405 as compared to budesonide. Finally, in vitro, RCD405 reduced the LPS-induced NF-κB activation and by itself reduced cellular metabolism.

**Conclusions:**

RCD405 has airway relaxant effects, and it reduces AHR as well as airway inflammation in the models used, suggesting that it could be a clinically relevant compound to treat inflammatory airway diseases. Possible targets of this compound are complexes of mitochondrial oxidative phosphorylation, resulting in decreased metabolic activity of targeted cells as well as through pathways associated to NF-κB. However, further studies are needed to elucidate the mode of action.

**Supplementary Information:**

The online version contains supplementary material available at 10.1186/s12931-024-02780-8.

## Introduction

Asthma is characterized by variable airflow obstruction, airway inflammation, and airway hyperresponsiveness (AHR) [1]. Clinically, the disease manifests as chest tightness, wheezing, coughing, and shortness of breath [2]. In allergic asthma, the predominating form of the disease, type 2 inflammation is seen (type 2-high asthma). This includes the release of cytokines, such as IL-4, IL-5, and IL-13, resulting in recruitment of eosinophils to the lungs [3]. The fundamental pharmacologic treatment for asthma patients relies on inhaled corticosteroids (ICS) and β2-adrenoceptor agonists. Despite a range of further treatment options such as inhaled muscarinic antagonists, oral corticosteroids, leukotriene receptor antagonists and humanized monoclonal antibodies (‘biologics’) against IgE and type 2 cytokines, a significant number of patients still suffer from poorly controlled asthma [4]. Some patients with type 2-high asthma experience treatment resistances to steroids and few treatment options exist for patients with type 2-low asthma where influx of neutrophils to the airways can be seen [5].

Chronic obstructive pulmonary disease (COPD) is a chronic airway disorder caused by inhalation of noxious particles or gases, including tobacco smoke. COPD is characterized by persistent respiratory symptoms and airflow limitation, as well as variable inflammatory patterns and tissue remodeling throughout the airways [6]. In COPD, there is in most cases, an inflammation dominated by neutrophils and both β2-agonists, muscarinic antagonists, and sometimes ICS are used to achieve bronchodilation [7]. However, type 2 inflammation and AHR are present in 20-50% of patients with COPD, and this is associated with an increased risk of exacerbations [8, 9]. In COPD, the frequency of exacerbations is tightly linked to morbidity and mortality [10]. The prevention of exacerbations is a major therapeutic aim in both asthma and COPD. The use of ICS, muscarinic antagonists, and phosphodiesterase inhibitors decreases the number of exacerbations but has marginal effect on mortality in COPD [7]. Thus, there is an unmet medical need for improved pharmacological intervention in both asthma and COPD.

Quinolines are one of the most ubiquitous heterocyclic moieties in medicinal chemistry with several beneficial chemical and pharmacological properties. Its scaffold is contained in many natural and synthetic compounds with biological activities such as antimalarial, anticancer, antifungal, antimicrobial, anti-inflammatory and anti-HIV properties [11, 12]. Herein, we present the novel quinoline RCD405 (the molecular structure can be found in Supplementary Fig. 1) and investigate its airway relaxing properties and anti-inflammatory effect in murine models of ovalbumin (OVA)-induced allergic asthma and LPS-induced airway inflammation. The LPS model was used to obtain an inflammatory response similar to that seen in COPD [13]. To further examine the mechanisms*,* the effects of RCD405 on LPS-induced NF-κB activation and metabolic activity were measured in the human monocytic cell line THP-1.

## Methods

### Ethical approval

All procedures involving human tissue were in accordance with the ethical standards of the relevant institution and/or national research committee of each collection site, REPROCELL’s ethical approval for use of human tissue (West of Scotland Research Ethics Service 17/WS/0049), and the amended Helsinki Declaration. Informed written consent was obtained from all individual participants or their nominated persons. Procedures involving rat and dog tissues were performed in accordance with the UK Animal Welfare Act 2006 and in collaboration with appropriate Home Office licensed facilities. All murine experiments were approved by the Malmö-Lund Animal Care Ethics Committee (ethical permit no. 3802-19) and the Stockholm Animal Care Ethics Committee (ethical permit no. 3649-2019). Female BALB/c mice (Janvier, Le Genest-Saint-Isle, France or Envigo, Horst, the Netherlands), 7-11 weeks old, were housed in plastic cages with absorbent bedding material, and maintained for 2 weeks before experimental initiation.

### Relaxation of human, canine, rat and mouse airways

Bronchi from humans and dogs, and trachea from rats and mice, were dissected free from the surrounding tissue, cut into rings (1-2 mm in length) and mounted in 5 mL myograph baths containing physiological saline solution, aerated with 95% O2 / 5% CO2 gas mix, warmed and maintained at approximately 37 °C throughout the experiment. The changes of smooth muscle force were detected using an isometric force-displacement transducer captured using Lab Chart software (ADInstruments, Sidney, Australia). Human, dog and rat airways were exposed to carbachol (10 μM for human; 1 μM for rats and dogs) followed by isoprenaline (10 μM) in order to assess their contractile and relaxant ability. Airways that did not respond to carbachol or isoprenaline were rejected. Mouse trachea was exposed to KCl (60 mM) twice (with a wash in between) to check the tissue viability. To investigate the relaxant effects of RCD405, a cumulative concentration response curve (CRC) performed on airways pre-constricted with carbachol (10 μM, human; 1 μM, dogs and rats; 0.3 μM, mouse). Concomitantly, the relaxant effects of the vehicle (DMSO) were defined as separate CRCs given at the same time point and at the same concentration as the dilution of RCD405. At the end of the CRC for human, dog and rat airways, theophylline was added to induce maximum relaxation which was used for normalization of the relaxation to RCD405. In the experiments with mouse trachea, the relaxation of RCD405 was normalized to the maximal contraction of the pre-contraction with carbachol and the viability of segments at the end of each experiment were confirmed by assessing the contraction to KCl (60 mM) and acetylcholine (1 mM). The potencies for each individual curve were calculated using non-linear fit with GraphPad Prism (GraphPad Software, San Diego, CA) giving an estimation of the pEC_50_ value (the negative logarithm of the EC_50_ value describing the concentration that causes half maximum effect).

### Study design of mouse experiments

To investigate the airway dilating and anti-inflammatory properties of RCD405 in vivo murine models of OVA-induced allergic asthma and LPS-induced airway inflammation were used. The OVA-model was chosen due to the resemblance of asthma pathology in humans, including AHR, excessive mucus production and eosinophilic airway inflammation. To reflect the inflammation of type 2-low asthma and some features of COPD, LPS-induced airway inflammation was applied [14]. To enable comparisons with drugs currently used for treatment of allergic asthma, mice treated with the corticosteroid budesonide were included.

### In vivo animal models

The mice were monitored and kept with controlled temperature and light/dark cycles and with food and water ad libitum. The induction of allergic airway inflammation was achieved by sensitization of all mice with 20 μg OVA in alum (1:10), dosed intraperitoneally on day 0 and 7, followed by intratracheal (i.t.) challenges with OVA (35 μg) on day 14, 16, 18, and 20 (Fig. 2A). RCD405 (10 mg/kg) or budesonide (3 mg/kg) were dosed i.t. 1 h prior to each challenge. The mice were sacrificed on day 21 and organs, plasma and bronchoalveolar lavage fluid (BALF) were collected. The mice were allocated randomly into five different experimental groups: PBS-challenged and treated with vehicle (Veh/PBS), PBS- challenged and treated with RCD405 (RCD405/PBS), OVA-challenged and treated with vehicle (Veh/OVA), OVA-challenged and treated with RCD405 (RCD405/OVA), OVA-challenged and treated with budesonide (BUD/OVA). For the AHR measurements, the budesonide group was not included. In the LPS model, airway inflammation was induced by an i.t. administration of LPS (3 mg/kg). RCD405 (10 mg/kg) was administered i.t. 1 h after LPS. The mice were sacrificed after 24 h of LPS administration. The mice were allocated into the following experimental groups: PBS challenged and treated with vehicle (Vehicle/PBS), PBS challenged and treated with RCD405 (RCD405/PBS), LPS challenged and treated with vehicle (Vehicle/LPS) and LPS challenged and treated with RCD405 (RCD405/LPS).

### Airway responsiveness measurements

Airway responsiveness was induced by administration of methacholine (MCh; Sigma-Aldrich) on day 21 in mice anaesthetized with ketamine hydrochloride (75 mg/kg, Ketaminol® Vet., Intervet, Stockholm, Sweden) and medetomidine hydrochloride (1 mg/kg, Cepetor®Vet., VETMEDIC, Stockholm, Sweden). Aerosol administration of methacholine was performed via a nebulizer (Scireq; Montreal, Canada), with doses of 0, 3.125, 6.25, 12.5, 25 and 50 mg/mL. A small animal ventilator (flexiVent; Scireq) was used to measure the airway responsiveness. The following measurements were recorded: dynamic resistance (Rrs), elastance (Ers), central resistance (Rn), peripheral tissue damping (G) and tissue elastance (H).

### Collection of murine blood

After euthanasia, blood was immediately collected by cardiac puncture in 0.1 M sodium citrate tubes (1:10 citrate:blood) and centrifuged at 1,000 × *g* for 10 min. Plasma supernatants were collected and used for the analysis of inflammatory mediators by a multiplex immunoassay (Bio-Plex; Bio-Rad, Hercules, CA).

### Collection of murine bronchoalveolar lavage fluid (BALF)

Bronchoalveolar lavage was performed with a total volume of 1 mL PBS containing 0.1 mM EDTA. BALF was collected in Eppendorf tubes on ice. The cells were spun down at 300 × *g* and the supernatants were transferred to -80 °C for multiplex immunoassay analysis. The spun down cells were resuspended in PBS and aliquoted for flow cytometry and cytospin differential counts. Cytospin preparations of cells were stained with May–Grünwald Giemsa stain (Histolab) and counted using light microscopy.

### Collection of murine lung tissue

Right lungs were collected in Eppendorf tubes containing RNA*later* (Thermo Fisher Scientific, Waltham, MA) and stored at -20 °C until used for real-time PCR. Frozen lungs were thawed and homogenized in tissue protein extraction reagent (T-PER) solution (Thermo Fisher Scientific) containing protease inhibitor (Pefabloc SC; Sigma-Aldrich) at a final concentration of 1 mM. Lung homogenates were centrifuged at 9,000 × *g* for 10 min at 4 °C, and the supernatants were collected for multiplex immunoassay (Bio-Plex; Bio-Rad) analysis. Left lungs were collected in Histofix (Histolab, Göteborg, Sweden) and submerged in 4% buffered paraformaldehyde solution followed by embedding in paraffin.

### Total IgE and OVA-specific IgE ELISAs

Total plasma IgE levels were determined using an IgE Mouse ELISA Kit (Invitrogen, Waltham, MA) according to the protocol provided by the manufacturer. The range of the assay was 0.137-100 ng/mL and the samples were diluted 1:500. OVA-specific IgE levels were measured using a LEGEND MAX™ Mouse OVA Specific IgE ELISA Kit (BioLegend, San Diego, CA) as per the manufacturer’s instructions. The range of the assay was 0.313-20 ng/mL and the samples were diluted 1:10.

### Flow cytometry

Flow cytometry was carried out using a BD Accuri C6 (BD, Franklin Lakes, NJ). Murine BALF cells were incubated with Fixable Viability Stain 510 (FVS510) (BD cat. #564406) to differentiate live and dead cells. This was followed by washing with Stain buffer (BD cat. #554656) and incubation with Lyse Fix (BD cat. #558049). Fixed cells were washed with stain buffer and aliquoted into two parts. One was incubated with CD11b (BD cat. #553312), CD11c (BD cat. #558079), Ly6G (BD cat. #551461) antibodies and the other aliquot was incubated with CD11c, MHC (BD cat. #558593), and SiglecF (BD cat. #562680) antibodies.

### Cell counting and cytospin preparations

Cells from BALF were counted using a Bürker-chamber and cytospin preparations were stained with May–Grünwald Giemsa to allow differential counting.

### H&E and PAS staining of murine lung sections

Right lungs were fixed in Histofix (Histolab) and embedded in paraffin. Sections (4 μm) were cut with a microtome and placed on glass slides (Superfrost Plus; Thermo Fisher Scientific). Deparaffinization was performed using serial dilutions of xylene and ethanol. Sections were stained with Alcian blue−periodic acid−Schiff (AB/PAS) staining. Briefly, sections were pretreated in 99.5% ethanol for 10 min, rinsed in running tap water for 10 min, and immersed in 3% acetic acid for 2 min. Staining with Alcian blue was carried out with 1% Alcian blue 8GX in 3% acetic acid (pH 2.5) for 15 min. Slides were then immersed in 3% acetic acid for 1 min and rinsed under running tap water for 9 min. Sections were oxidized in 1% periodic acid for 10 min,

rinsed under running tap water for 5 min, immersed in 25% Schiff’s reagent (Sigma-Aldrich, St. Louis, MO), and rinsed under running tap water for 5 min. Slides were treated with 0.5% sodium metabisulfite three times for 1 min, washed with tap water, dehydrated, and mounted. The sections wer also stained with Mayer hematoxylin and 0.2% eosin (H&E). H&E and AB/PAS stained sections were scanned with a slide-scanning robot (ScanScope Slide Scanner, Aperio Technologies). Computer-assisted image analysis was performed using the Aperio ImageScope software (Leica Biosystems). To score inflammation of the H&E stained slides, we used an algorithm that measured the total area (μm^2^) of inflammatory cell lesions in each tissue sections. An algorithm of positive pixel counting was used for the quantification of the percentage of AB/PAS positive cells in the airways.

### Cytokine analysis

For the detection of multiple cytokines in BALF, plasma, and lung homogenate, the Bio-Plex Pro mouse cytokine assay (23-Plex Group I; Bio-Rad) was used on a Luminex-xMAP/Bio-Plex 200 System with Bio-Plex Manager 6.2 software (Bio-Rad). The detection ranges were as follows: Eotaxin (1.15-21372.02 pg/mL), GCSF (6.97-124018.4 pg/mL), GMCSF (3.73-1161.99 pg/mL), IFN-γ (0.72-14994.64 pg/mL), IL-1α (0.63-10337.5 pg/mL), IL-1β (1.57-28913.54 pg/mL), IL-2 (1.21-22304.34 pg/mL), IL-3 (0.47-7639.21 pg/mL), IL-4 (0.36-6334.86 pg/mL), IL-5 (0.76-12950.39 pg/mL), IL-6 (0.66-11370.16 pg/mL), IL-9 (2.46-2580.93 pg/mL), IL-10 (4.09-76949.87 pg/mL), IL-12p40 (17.38-323094.58 pg/mL), IL-12p70 (19.51-79308.46 pg/mL), IL-13 (53.85-257172.3 pg/mL), IL-17 (0.5-8355.61 pg/mL), KC (1.3-23377.88 pg/mL), MCP-1 (45.04-223776.6 pg/mL), MIP-1α (0.58-14038.07 pg/mL), MIP-1β (2.39-928.18 pg/mL), RANTES (4.42-4721.74 pg/mL), and TNF-α (4.61-73020.1 pg/mL). Cytokine measurements for samples were corrected for protein concentration. These were determined using a Pierce™ BCA Protein Assay Kit (Thermo Fischer Scientific).

### Real-time PCR array

Extraction of total mRNA from lung tissue submerged in RNA*later* was performed using an RNeasy Mini Kit (Thermo Fischer Scientific) in accordance with the protocol from the manufacturer. The concentrations of RNA in the samples were determined with a NanoDrop ND1000 (Saveen Werner). RNA was pooled in equal amounts from each animal in the experimental groups (Veh/PBS *n* = 8, Veh/OVA *n* = 8, RCD405/OVA *n* = 8 and BUD/OVA *n* = 8). An iScript Advanced cDNA Synthesis Kit (Bio-Rad) was used to synthesize cDNA. SYBR® Green ROX™ qPCR Mastermix was added to the cDNA and the samples were transferred to all wells of a RT^2^ Profiler™ PCR Array Mouse Allergy & Asthma PAMM-067ZA plate. A QuantStudio™ 7 Flex system (Thermo Fisher Scientific) was used for the RT-PCR reaction and the data was analyzed with the manufacturer’s web-based software (https://geneglobe.qiagen.com/analyze). Gene expression was normalized to house-keeping genes *B2m*, *Actb*, *Gusb*, *Gapdh* and *Hsp90ab1*. Genes with a fold regulation above 2.5 were arbitrarily defined as biologically significant and further compared between the treatment groups.

### Cell culture and NF-κB activation assay

THP1-Blue™ cells (Invivogen, San Diego, CA, USA), that are derived from the human THP-1 monocyte cell line by stable integration of an NF-κB-inducible secreted embryonic alkaline phosphatase (SEAP) reporter construct, were grown in T75 flasks with RPMI supplemented with 10% FBS, 1% antibiotic–antimycotic, 0.25% G418 and 0.2% phleomycin D1. Cells were seeded (1 × 10^5^ per well) and stimulated with LPS (1 ng/mL) and RCD405 (0.5, 5 or 50 μM) followed by incubation for 24 h at 37 °C in 5% CO_2_. Cells were collected to detect NF-κB activation, mitochondrial reductase activity (as detected by MTT conversion to formazan), and LDH release assays. In a 96-well plate, 180 μL QUANTI-Blue™ solution was added to each well, and subsequently 20 μL medium from the cell experiments was transferred to the wells. The plate was incubated at 37 °C for ~ 1 h and optical density was measured at 595 nm using a VICTOR 1420 Multilabel plate reader.

### MTT assay and measurement of LDH release

Mosmann’s MTT [3-(4,5-dimethylthiazol-2-yl)-2,5-c] assay was utilized to detect cell metabolic activity in THP-1-Blue™ cells. MTT solution was added to a final concentration of 0.5 mg/mL in all wells and the plate was incubated for 1,5 h at 37 °C. The plates were centrifuged at 1000 rpm for 5 min. The supernatant was removed from the wells and 100 μL DMSO was added followed by shaking for 5 min. The absorbance of the MTT dye was measured at 550 nm using a VICTOR 1420 multilabel reader (PerkinElmer). LDH release was measured in the cell media using a CytoTox 96® Non-Radio Cytotoxicity Assay (Promega, Madison, WI) according to instructions from the manufacturer. Briefly, 50 μL aliquots of cell media from the cell experiments were transferred to a 96 well plate. A volume of 50 μL CytoTox 96® reagent was added to each well and the plate was incubated in dark for 30 min at room temperature. The reaction was stopped by adding 50 μL Stop Solution and the absorbance was measured at 490 nm (VICTOR 1420 multilabel reader, PerkinElmer).

### Statistical analysis

Analyses of differences between three or more groups were calculated using one-way ANOVA with Šídák’ post hoc test or two-way ANOVA with Šídák’s multiple comparisons test. The calculations were made using GraphPad Prism (GraphPad Software). Statistical significance was defined as *P* < 0.05. The results are shown as mean ± SEM.

## Results

### RCD405 has a relaxant effect on carbachol pre-contracted airways isolated from humans, dogs, rats and mice without binding to muscarinic or β2-adrenergic receptors

**Fig. 1:**
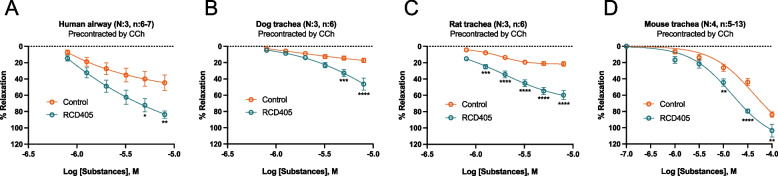
The relaxing effect of RCD405 on human, dog, rat and mouse isolated airway pre-contracted with CCh. Concentration-response relaxation curves for RCD405 and vehicle controls (DMSO) after pre-contraction with CCh in isolated airways from (**A**) human, (**B**) dog, (**C**) rat and (**D**) mouse. Differences between groups were analyzed using a Two-way ANOVA followed by Šídák's multiple comparisons test (*P ≤ 0.05, **P ≤ 0.01, ***P ≤ 0.001, ****P ≤ 0.0001). N refers to number of individuals and n to number of segments

In the isolated airway from all species tested, RCD405 showed acute concentration-dependent relaxations of the CCh pre-contracted segments (Fig. 1A-D). At the highest concentrations of RCD405 for all CRCs, the relaxations were significantly different compared to the vehicle controls (DMSO) and the maximal relaxation for human, dog, rat and mouse airways were 39 ± 11%, 29 ± 17%, 38 ± 13% and 39 ± 12% respectively, when normalized to the effect of DMSO. When defining the potencies with the non-linear fit, the pEC_50_ values for RCD405 were 5.9 ± 0.3, 5.0 ± 0.1, 5.8 ± 0.1 and 4.7 ± 0.1, representing EC50 values of 1.3, 10, 1.6 and 20 μM, in human, dog, rat and mouse airways, respectively.

### RCD405 administration dampens OVA-specific IgE and airway hyperresponsiveness

**Fig. 2:**
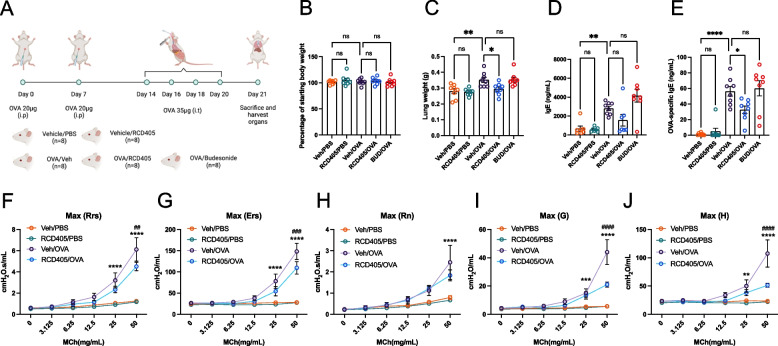
The effect of RCD405 treatment on body weight, lung weight, IgE levels and airway hyperresponsiveness. **A** Experimental setup of the OVA-induced allergic airway inflammation model. Mice were divided into the following experimental groups: Veh/PBS (*n* = 8), RCD405/PBS (*n* = 8), Veh/OVA (*n* = 8), RCD405/OVA (*n* = 8), and BUD/OVA (*n* = 8). Each mouse received an intraperitoneal injection of 20 μg OVA in alum (1:10) at day 0 and 7. Intratracheal challenges with 35 μg OVA were performed at day 14, 16, 18 and 20 in the Veh/OVA, RCD405/OVA, and BUD/OVA groups. Mice were treated with RCD405 (10 mg/kg) or budesonide (3 mg/kg) intratracheally 1 h before each challenge. **B** Mouse weights on day 21 shown as percentage of starting weight. **C** Lung weights on day 21. **D** Total IgE and (**E**) OVA-specific IgE levels measured by ELISA in plasma from Veh/PBS, RCD405/PBS, Veh/OVA, RCD405/OVA, and BUD/OVA treated mice. Using a flexiVent, several different parameters were measured: (**F**) Rrs, dynamic resistance, assessing the level of constriction in the lungs, (**G**) Ers, elastance, representing the stiffness of the respiratory system, (**H**) Rn, Newtonian resistance, representing the resistance of conducting airways, (**I**) G, tissue damping, reflecting tissue resistance, and (**J**) H, tissue elastance. Experimental groups: Veh/PBS (*n* = 10), RCD405/PBS (*n* = 10), Veh/OVA (*n* = 9) and RCD405/OVA (*n* = 7). Statistical comparisons were made using one-way ANOVA or Two-way ANOVA followed by Šídák's multiple comparisons test (*P ≤ 0.05, **P ≤ 0.01, ***P ≤ 0.001 and ****P ≤ 0.0001, Veh/OVA vs Veh/PBS and RCD405/PBS groups. ##*P* ≤ 0.01, ###*P* ≤ 0.001, and ####*P* ≤ 0.0001, Veh/OVA vs RCD405/OVA)

To investigate the effect of RCD405 on airway hyperresponsiveness, an OVA mouse model was used (Fig. 2A) with or without RCD405 intervention. The weights of mice, shown as percentage of starting weights, were not affected by the sensitization or challenges (Fig. 2B). A slight increase in lung weights could be found in the Veh/OVA group compared with Veh/PBS (Fig. 1C), which could not be seen in the RCD405/OVA group. The total IgE level was significantly increased in the Veh/OVA group compared to Veh/PBS control (Fig. 2D) which was not altered by budesonide. The decrease of total IgE level in the RCD405/OVA group, compared to Veh/OVA, failed to reach statistical significance. Compared to Veh/PBS control, the level of OVA-specific IgE was markedly increased in the Veh/OVA group (Fig. 2E). Though not altered by budesonide, the increase of OVA-specific IgE was decreased by RCD405 interventions.

The airway responsiveness to methacholine (MCh) was measured in flexiVent and showed that animals challenged with PBS demonstrated a weak concentration dependent reaction to MCh in all the parameters (*i.e.*, dynamic resistance: Rrs; elastance: Ers; Newtonian resistance: Rn, tissue damping: G and tissue elastance: H) (Fig. 2F-J). No significant differences were found in control animals that were pre-treated with either RCD405 or vehicle. In contrast, OVA-exposed animals showed exaggerated MCh responses in all of the above-mentioned measurements. Pre-treatments with RCD405 significantly dampened the reactions for the whole lung described in Rrs (Fig. 2F) and Ers (Fig. 2G), and as well in the peripheral lung described in G (Fig. 2I) and H (Fig. 2J), whereas the difference in Rn which is the effect in the conducting airways (Fig. 2H) did not reach statistical significance.

### RCD405 administration reduces immune cell recruitment to the lungs

**Fig. 3:**
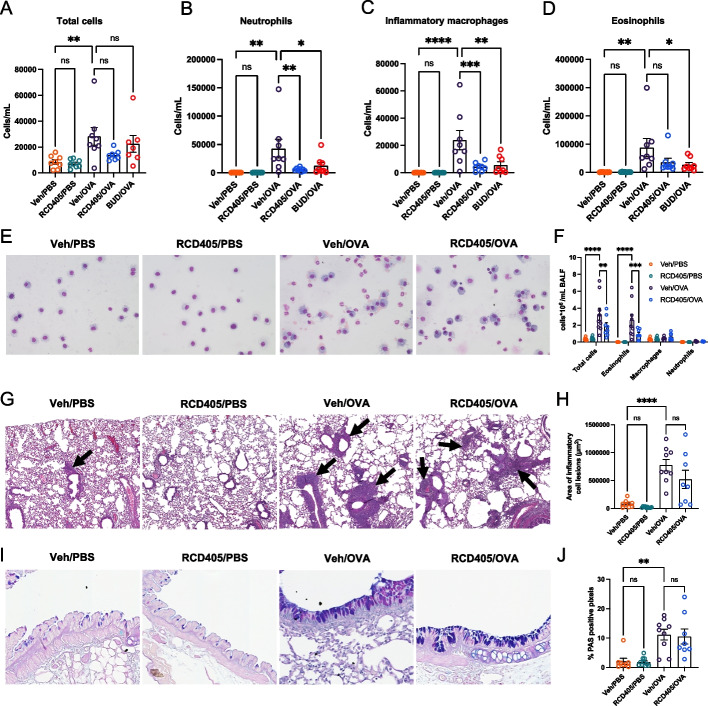
Immune cell recruitment and lung histology. **A** Total cell, (**B**) neutrophil, (**C**) inflammatory macrophage and (**D**) eosinophil concentration in BALF of Veh/PBS (*n* = 8), RCD405/PBS (*n* = 8), Veh/OVA (*n* = 8), RCD405/OVA (*n* = 7) and BUD/OVA (*n* = 7) treated mice measured by flow cytometry. **E** Representative Giemsa-Wright stained cytospins of BALF samples from Veh/PBS, RCD405/PBS, Veh/OVA and RCD405/OVA. **F** Differential counts of total cells, eosinophils, macrophages, and neutrophils in Giemsa-Wright stained cytospins from BALF samples. **G** H&E and (**I**) AB/PAS staining on 4 μm lung tissue sections from mice with the following treatments: Veh/PBS (*n* = 9), RCD405/PBS (*n* = 10), Veh/OVA (*n* = 9) and RCD405/OVA (*n* = 8). **H** Areas (μm^2^) of inflammatory cell lesions (black arrows) were measured with ImageScope software. **J** Mucus production was analyzed by calculating the PAS % of positive pixels in the bronchi with ImageScope software. Statistical comparisons were performed using one-way ANOVA followed by Šídák's multiple comparisons test. (ns: not significant, (*P ≤ 0.05, **P ≤ 0.01, ***P ≤ 0.001 and ****P ≤ 0.0001)

Flow cytometry analysis of BALF samples was used to investigate recruitment of immune cells to the lungs after the induction of allergic airway inflammation. A large increase in the total number of cells was seen in the Veh/OVA group compared to the two control groups, Veh/PBS and RCD405/PBS, respectively (Fig. 3A). Administration of RCD405 or budesonide resulted in a reduction of recruited immune cells but failed to reach statistical significance. When analyzing the individual immune cell types, significant decreases in neutrophils and inflammatory macrophages were seen in the RCD405 and budesonide treated animals (Fig. 3B and C). Additionally, recruitment of eosinophils to the lungs was reduced by budesonide, whereas RCD405 showed a non-significant trend towards a reduction (Fig. 3D).

An increase in total immune cells in the Veh/OVA group was observed, which was significantly reduced by RCD405 treatment (Fig. 3E and F). The immune cells mostly consisted of eosinophils and the RCD405/OVA group had significantly fewer eosinophils compared to the Veh/OVA group. No differences were found in macrophage and neutrophil counts between any of the groups (Supplementary Fig. 2).

### RCD405 administration does not decrease the OVA-induced inflammation or mucin production in mouse airways

The total area of inflammatory cell lesions detected by H&E staining were elevated in the OVA-challenged mouse airways compared to the controls (Veh/OVA vs Veh/PBS and RCD405/OVA vs RCD405/PBS) whereas there was no difference between the RCD405 treated and control groups (RCD405/OVA vs Veh/OVA) (Fig. 3 G and H). AB/PAS staining revealed increased mucus production in the lungs of OVA-challenged mouse groups compared to the controls, however, no differences in mucus production were found between the RCD405 treated and vehicle treated groups (Fig. 3I and J). Images of whole lung sections can be found in Supplementary Fig. 8 and 9.

### RCD405 administration reduces cytokine and chemokine production in the inflamed lung

**Fig. 4:**
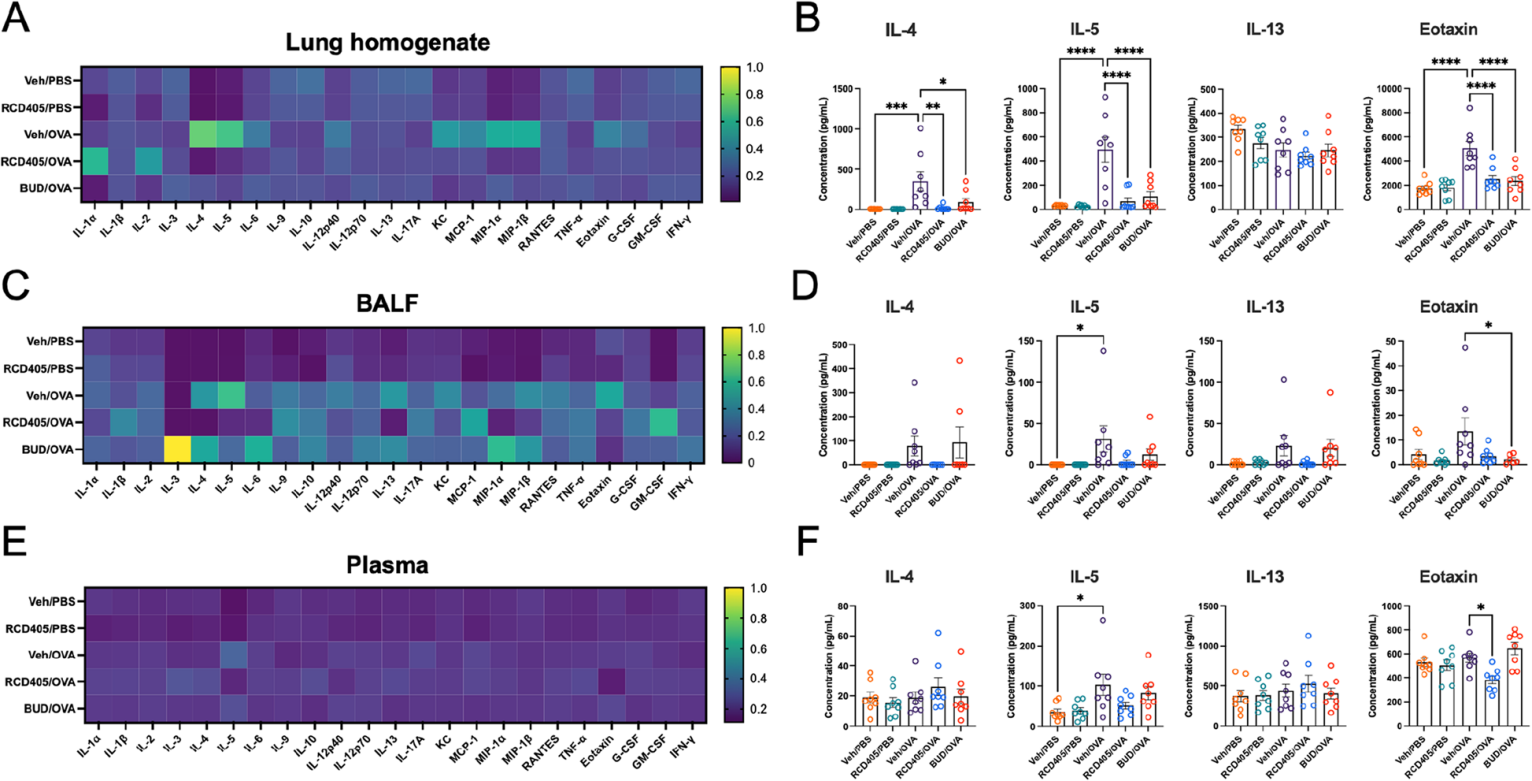
Cytokine and chemokine levels in lung homogenate, BALF, and plasma. **A**, **C** and **E** Heatmaps showing differences in cytokine and chemokine levels in lung homogenates, BALF and plasma, where yellow indicates a high value and dark blue indicates a low value. **B**, **D** and **E**) Individual graphs with the type 2 immunity associated proteins IL-4, IL-5, IL-13, and eotaxin shown with statistical comparisons. Experimental groups: Veh/PBS (*n* = 8), RCD405/PBS (*n* = 8), Veh/OVA (*n* = 8), RCD405/OVA (*n* = 8), and BUD/OVA (*n* = 8). All individual cytokine graphs can be found in Supplementary Fig. 2–4. Differences between groups were analyzed using a one-way ANOVA followed by a Šídák's post hoc test (*P ≤ 0.05, **P ≤ 0.01, ***P ≤ 0.001, ****P ≤ 0.0001)

Using a 23-cytokine multiplex immunoassay, proinflammatory cytokines and chemokines were measured in lung homogenate, BALF, and plasma (Fig. 4A-F). In lung homogenate, the type 2 related cytokines/chemokines, namely IL-4, IL-5 and eotaxin, were significantly increased in Veh/OVA group. These increases were significantly reduced in RCD405/OVA and budesonide/OVA treated mice compared to Veh/OVA (Fig. 4A and B). Additionally, significant reductions after RCD405 treatment were seen in IL-6, KC, MCP-1α, MIP-1β.

In BALF samples, IL-5 was significantly increased in the Veh/OVA group compared to Veh/PBS. However, the increase of all other cytokines/chemokines and the effect of RCD405 on those cytokines/chemokines failed to reach statistical significance (Fig. 4C and D). The increased level of IL-5 could be found in plasma of animals from Veh/OVA group compared to Veh/PBS, though no other cytokines/chemokines were found altered (Fig. 4E and F). Individual graphs of all cytokines and chemokines tested in BALF, plasma and lung homogenates can be seen in Supplementary Fig. 2-4.

### RCD405 administration results in decreased expression of genes associated with type 2 inflammation

**Fig. 5:**
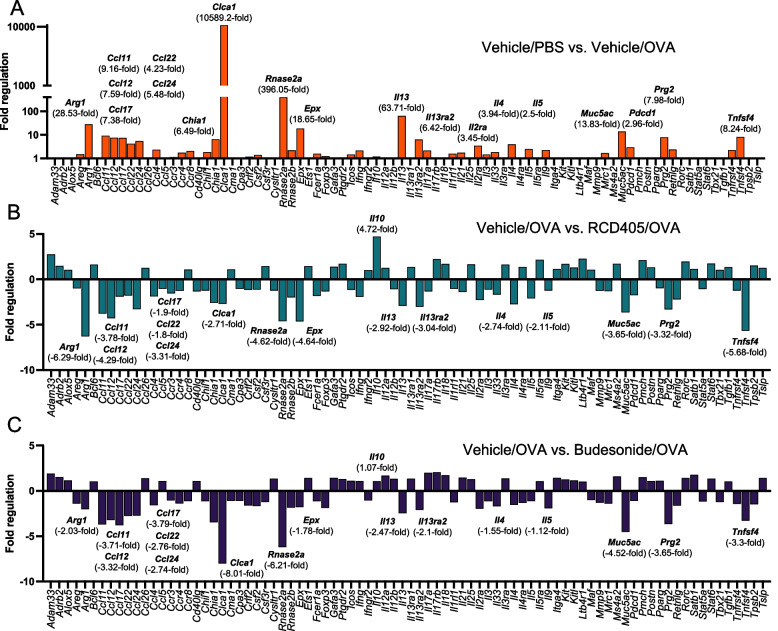
Expression profiling of genes related to asthma and allergy in murine lung tissue. The gene expression of pooled lung tissue mRNA from (**A**) PBS challenged and vehicle treated mice (Vehicle/PBS) compared with OVA challenged and vehicle treated mice (Vehicle/OVA) on day 21. Genes with a fold regulation above 2.5 is highlighted. **B** Mice challenged with OVA and treated with vehicle compared with mice challenged with OVA and treated with RCD405. Genes with more than a 2.5 fold increase in fold regulation in (**A**) are highlighted. **C** Mice challenged with OVA and treated with vehicle compared with mice challenged with OVA and treated with budesonide. Genes with more than a 2.5 fold increase in fold regulation in (**A**) are highlighted. Equal amounts of mRNA were pooled from 8 animals in each group

A PCR-array including 84 genes involved in allergy and asthma, was applied on total mRNA from murine lung tissue. Expression profiles of Veh/PBS, Veh/OVA, RCD405/OVA and BUD/OVA were normalized to five house-keeping genes. To evaluate the effect of the OVA induced airway inflammation, fold regulation values for all genes were generated by comparing the expression profiles of Veh/PBS and Veh/OVA, which showed a large increase in expression of several genes (Fig. 5A). To further elucidate the treatment effect of RCD405 and budesonide, comparisons were made between Veh/OVA and RCD405/OVA (Fig. 5B) or BUD/OVA (Fig. 5C) where genes with a 2.5 increase in fold regulation were chosen to be relevant. The gene with the most prominent increase in expression was *Clca1*, which encodes the mucus production regulator Clca1 (calcium-activated chloride channel regulator 1). The expression was reduced upon treatment with both RCD405 (-2.71) and budesonide (-8.01). Increased expression was also found in the case of several other genes in the OVA treated mice compared to the controls, including the type 2 immunity related genes *Il-4*, *Il-5* and *Il-13* (Fig. 5A). Treatment with RCD405 resulted in a decreased expression of *Il-4* (-2.74), *Il-5* (-2.11) and *Il-13* (-2.92). Treatment with budesonide also reduced the expression of the type 2 related genes, but to a lesser extent than RCD405. Other genes with a noticeable downregulation after drug treatment included the macrophage M2-marker *Arg1* (RCD405 [-6.29], budesonide [-2.03]), the T cell activator *Tnfsf4* (RCD405 [-5.68] and budesonide [-3.3] and the goblet cell produced mucin *Muc5ac* (RCD405 [-3.65] and budesonide [-4.52]). Additionally, OVA-induced expression of the eosinophil granulocyte proteins *Rnase2* and *Epx*, was reduced by treatment with RCD405 (-4.62) and budesonide (-6.21). Finally, an increased expression was found for the anti-inflammatory cytokine *Il-10* (4.72) in the RCD405 treated mice compared to the Veh/OVA mice, which was not seen in the budesonide group (1.07).

### LPS induced airway inflammation is reduced after administration of RCD405

**Fig. 6:**
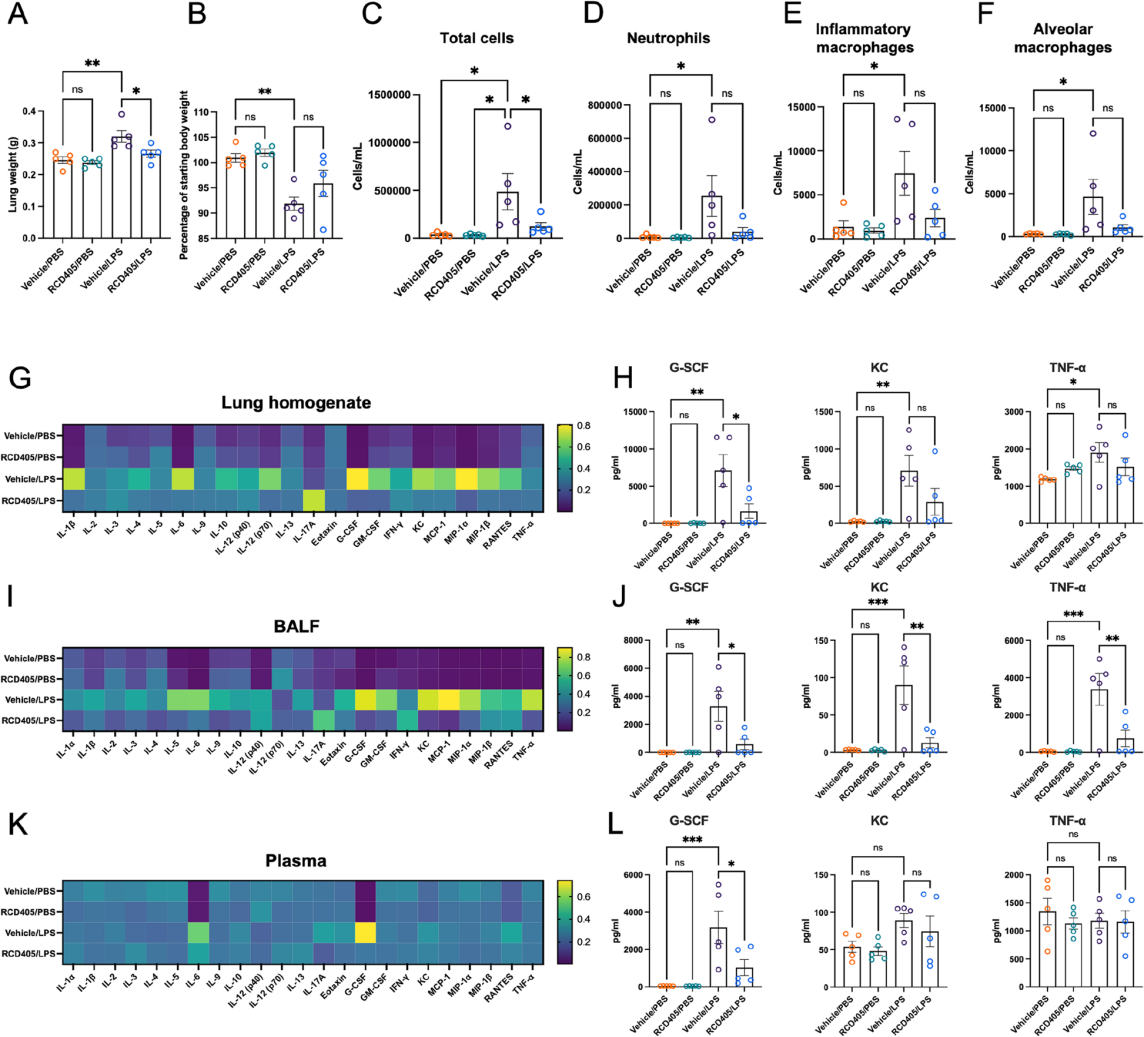
The effect of RCD405 treatment in LPS induced airway inflammation. Experimental groups: Veh/PBS (*n* = 5), RCD405/PBS (*n* = 5), Veh/LPS (*n* = 5), RCD405/LPS (*n* = 5). **A** Murine lung weights recorded on day 21. **B** Murine body weights at day 21 shown as percentage of starting weight. Total cells (**C**), neutrophils (**D**), inflammatory macrophages (**E**) and alveolar macrophages (**F**) in BALF at day 21. **G**, **I** and **K** Heatmaps showing cytokine and chemokine levels in lung homogenate, BALF and plasma. **H**, **J** and **L** Individual graphs of COPD related cytokines G-CSF, KC, and TNF-α. All individual cytokine graphs can be found in Supplementary Fig. 5–7. Statistical comparisons were performed using a one-way ANOVA followed by a Dunnett’s post-hoc test (*P ≤ 0.05, **P ≤ 0.01, ***P ≤ 0.001, ****P ≤ 0.0001)

The LPS model was used to obtain an inflammatory response similar to that seen in COPD. Even if LPS can cause bronchial hyperreactivity, this is not a key feature of COPD [13]. In the LPS model, the lung weights from the Vehicle/LPS group were significantly higher than control animals receiving PBS (Vehicle/PBS). However, the treatment with RCD405 significantly reduced the weight increase induced by LPS (Fig. 6A). The body weight was decreased after 24 h of LPS exposure (Vehicle/LPS) compared to control, whereas no significant drop of body weight could be found in the LPS exposed animals after RCD405 treatments (RCD405/LPS) (Fig. 6B).

Flow cytometry analysis of BALF revealed an increase in total cells, neutrophil, inflammatory macrophage, and alveolar macrophage numbers in the LPS treated group, whereas administration of RCD405 showed a trend towards a decreased number of all three cell types (Fig. 6C).

Cytokine analysis of lung homogenate, BALF, and plasma revealed large differences between the experimental groups (Fig. 6G-L). An increased expression of the COPD related cytokines G-CSF, KC and TNF-α was seen in lung homogenate (Fig. 6G and H) and BALF (Fig. 6I and J) in the LPS group, which was decreased substantially by the RCD405 treatment. In plasma, LPS induced a large increase in G-CSF which was significantly reduced upon RCD405 treatment (Fig. 6K and L). Several other key cytokines involved in airway inflammation were significantly decreased by RCD405 treatment, including MIP-1α and MCP-1. Individual differences for each cytokine tested, including statistical comparisons, can be seen in Supplementary Fig. 5–7.

### Inhibition of LPS-induced NF-κB, reduction of metabolic activity and increased release of LDH by RCD405

**Fig. 7:**
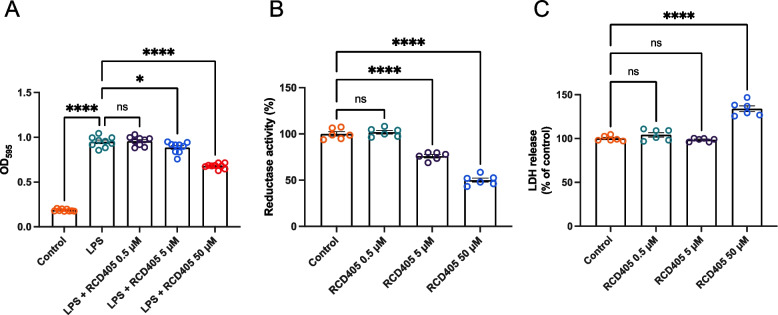
Effect of pre-incubation with RCD405 on LPS induced NF-κB signaling, metablic (reductase) activity, and LDH release in THP1-Blue cells. **A** NF-κB signaling in LPS-stimulated cells in the absence or presence of RCD405 at the indicated concentration. **B** Reductase-activity as measured by an MTT assay in THP1-Blue cells incubated with RCD405 at the indicated concentrations for 24 h. **C** Cytotoxicity as measured by release of LDH in THP1-Blue cells incubated with the indicated concentrations of RCD405 for 24 h. Statistical comparisons were performed using a one-way ANOVA followed by a Dunnett’s post-hoc test, (*P ≤ 0.05, **P ≤ 0.01, ***P ≤ 0.001, ****P ≤ 0.0001). Experiments were performed in triplicates and repeated three times (*n* = 9)

NF-κB is a key transcription factor, inducing transcription of a broad range of proinflammatory cytokines and chemokines [15]. To investigate if RCD405 affected the activation of this pathway, the human monocytic cell line THP-1 (transfected with a plasmid containing an NF-κB sensitive reporter gene) was preincubated with RCD405 before adding LPS (1 ng/mL) for 24 h. RCD405 caused a concentration-dependent reduction of NF-kB activation (Fig. 7A). In separate experiments, RCD405 showed a dose-dependent reduction of metabolic activity in THP-1 cells as measured by an MTT assay, reflecting NAD(P)H-dependent oxidoreductase enzyme activity [13] (Fig. 7B). Additionally, in THP-1 cells, the cytotoxicity of RCD405 was measured using an LDH assay. No significant changes of LDH release were found with low concentrations of RCD405 (*i.e.,* 0.5 μM and 5 μM; Fig. 7C). However, an increased release of LDH was seen at the highest concentration of RCD405 (50 μM), indicating cell-death.

## Discussion

This study demonstrates that RCD405 has acute relaxant effects in airways from different species and inhibits chronic OVA-induced and acute LPS-induced inflammation. For the OVA-induced allergic airway inflammation, RCD405 reduced both the induction of airway hyperresponsiveness and the influx of immune cells into the lung. RCD405 also reduced the levels of proinflammatory cytokines and chemokines in BALF and lung homogenate in both OVA- and LPS-induced airway inflammation. In addition, RCD405 reduced the LPS-induced NF-κB activation and the cellular metabolism in monocytic cells in vitro.

The results from the isolated airways indicated a general acute relaxant property of RCD405 as the effects were concentration-dependent in four different species including humans. Although the analyses of the potencies may not be totally accurate as the CRC did not always reach plateaus, the ranking of the potencies for RCD405 was human = rat > dog = mouse (*P* < 0.05) when compared between the species. However, the potency was markedly lower than for formoterol, salmeterol and terbutaline comparing the response in human bronchi [16] and mouse trachea [17]. The maximal effect of RCD405 was also complicated to define since the highest concentrations of RCD405 were not used in all experiments and that the vehicle with DMSO caused a relaxing effect by itself. When taking the effect of the vehicle control into consideration, the maximal effect of RCD405 was also lower in human bronchi and mouse trachea compared to the maximal effect for the β2-adrenergic receptor agonists which all reached almost maximal relaxation in the same tissues [16, 17]. Since RCD405 did not bind to the β2-adrenergic receptor nor the M1, M2 and M3 receptors (Supplementary Fig. 10), neither antagonistic effect against the carbachol pre-contraction nor activating relaxation through the β2-adrenergic pathway could be the reason for the relaxation induced by RCD405. Similar conclusion has been drawn from a previous study with an analogue to RCD405 [18]. In the same study, activation of the NO-pathway and transient receptor potential vanilloid (TRPV) channels were also excluded. An even more extensive investigation of the airway relaxing mechanisms for another quinoline, imiquimod, showed that it was independent of nitric oxide, carbon monoxide, and cAMP signaling, as well as neuronal activity [19]. A small effect of prostanoids was found in the same study indicating a release from the epithelial layer. This may be of relevance since the strongest effect in vivo of RCD405 was shown in the distal airways which have a high number of epithelial cells compared to smooth muscle cells. On the other hand, imiquimod induced intracellular calcium ([Ca2 +]i) release in cultured human smooth muscle cells that inhibited histamine-induced ([Ca2 +]i) release [19], indicating a similar mechanism as for chloroquine that caused a localized ([Ca2 +]i) response leading to smooth muscle membrane hyperpolarization [20]. However, whether the same mechanism is applicable for RCD405 needs to be further investigated. Moreover, additional investigations regarding pharmacokinetic and pharmacodynamic characteristics of RCD405 should be performed to determine the local concentrations and clinical doses needed for establishing relevant effects.

As predicted from previous studies, repeated exposures to OVA induced AHR to methacholine in mice, both in the central and peripheral lung, together with an increase of eosinophils in the BAL fluid and total IgE in the serum [21]. Intratracheal administration of RCD405 before each OVA-challenge reduced AHR, especially in the peripheral lung, reflected by significantly dampened reactions in G and H responses. A decrease in both IgE and AHR has also been shown to be caused by two other quinolines, chloroquine (50 mg/kg) and quinine (10 mg/kg), in a similar mouse model of OVA induced allergic airway inflammation [22] although different parts of the lung function were not evaluated. Thus, the present data with RCD405 are supported by similar experiments with other quinolines.

Regarding the effect on cellular influx in BALF, RCD405 had no significant effect on eosinophils in the OVA-model, while it reduced the influx of neutrophils and macrophages to the lung in the OVA model and showed a trend towards a decrease in the LPS model. That inflammatory immune cells in BALF differ from cells in the lung have previously been shown [23]. The cytokines/chemokines prototypic of allergic inflammation, i.e. IL-4, IL-5, and eotaxin, were all reduced in lung homogenate, demonstrating a strong inhibitory effect on type 2 inflammation by RCD405. This was also further supported by the RT-PCR data showing a decreased expression of *Il4*, *Il5* and *Ccl11* (eotaxin) in lung tissue from RCD405 treated mice. The reduction of IL-13 in lung homogenate, another Th2-dependent cytokine and inducer of IgE and eosinophils, was however not significant. Since IL-13 is suggested to modulate goblet cell differentiation or activation, its limited decrease might thus explain the apparent lack of effect on inflammatory cell lesions detected by H&E staining as well as mucus production after RCD405 dosing [24].

On a gene-expression level, several cytokines/chemokines were up-regulated in the Veh/OVA group compared to the Veh/PBS group, where *Clca1* displayed the most distinct difference with a 10589.2-fold increase. Clca1 encodes the protein Chloride channel accessory 1 which regulates mucus production in goblet cells [25]. RCD405 treatment resulted in only a small reduction in the expression of *Clca1* (-2.71-fold), potentially further explaining RCD405’s lack of effect on mucus production. Additionally, RCD405 caused a strong reduction in *Arg1* expression with a -6.29-fold decrease. Arginine metabolism is important in type 2 high airway inflammation and asthma, generating NO by several enzymes, including inducible nitric oxide synthase (iNOS), arginase-1 (ARG1), and ARG2 [26]. While ARG2 protects against allergic airway inflammation, roles for ARG1 seem more complex [27]. In mouse models of allergic asthma, deletion of ARG1 did not have any effect in some studies but attenuated airway inflammation in female mice in other, indicating a gender difference [28–30]. Thus, the suppression of *Arg1* by RCD405 in the current model, using female BALB/c mice may be important but the translation to humans remains to be investigated. It is also interesting to note that *Il10* gene expression is upregulated during treatment with RCD405 with a 4.72-fold increase. This cytokine is ascribed several immunosuppressive roles in allergic inflammation [31].

In this study the experimental design involving THP-1 cells transfected with an NF-κB sensitive reporter gene provided a controlled platform to investigate the effects of RCD405 on NF-κB activation. The observed dose-dependent reduction in NF-κB activation strongly indicates RCD405's potential as a regulator of the proinflammatory response in the context of LPS-induced airway inflammation [15]. In THP-1 cells, using an MTT assay, RCD405 showed a decrease in metabolic activity of the mitochondrial system. In mitochondria, the oxidative phosphorylation (OXPHOS) is executed by five respiratory complexes, i.e. complex I, II, III, IV, and V. In recent years, complex II (SHD) of the mitochondrial OXPHOS has caught increasing attention as an important regulator of inflammation where itaconate, which is derived from citric acid of the Kreb’s cycle, is an inhibitory factor in myeloid cells [32]. In vitro studies have shown that RCD405 possesses inhibitory effects on components of the mitochondrial OXPHOS system (Supplementary Fig. 11). The decreased metabolism caused by RCD405 in the monocytic cell line THP-1, was reflected in the MTT assay. Thus, the airway relaxation and anti-inflammatory effects of RCD405 may, at least in part, be mediated through inhibition of the respiratory complexes. Furthermore, mitochondria are crucial for airway smooth muscle function through their generation of ATP and in processes important to airway contractility, such as generation of reactive oxygen species (ROS) and roles in calcium homeostasis [33]. It is therefore likely that RCD405 interference with mitochondrial function could impair smooth muscle contraction, resulting in bronchodilation. By studying LDH release from THP-1 cells, it was shown that RCD405 only caused an increase at the highest concentration, which can indicate cytotoxicity. However, it is not likely that any toxic effect was induced during the in vivo studies, since the dose of RCD405 that both induced marked anti-inflammatory effects in both the OVA or the LPS models caused no weight loss of the mice. Further studies to investigate potential unwanted or toxic effects are being conducted. Except for a slightly lower body weight gain at the highest dose of 21 mg/kg, as compared to control animals, no other findings were reported. Clinical pathology and histopathology assessments were unaffected by the treatment.

In a wider perspective, RCD405 could have the potential of reducing the need for ICS and more important, oral corticosteroids in asthma and COPD. Quinolines are a large group of N-based heterocyclic aromatic compounds, with many having bioactive properties including pharmacological effects in several diseases [34]. Some alkaloids of this group have shown inhibitory activities of the proinflammatory transcription factors nuclear factor of activated T-cells (NFAT) and NF-κB [35]. Other activities from members of this group of compounds include inhibition of LPS-induced NO production, reduced activities of COX-2 and 5-LOX, reduced neutrophil infiltration as well as decreased oxidative stress at sites of inflammation [36, 37]. However, to the best of our knowledge, no quinoline has hitherto been investigated in the context of asthma or COPD.

The most used drugs for asthma treatment are ICS, and therefore budesonide was included as a comparator drug in the OVA model. RCD405 and budesonide showed similar effects in most readouts, but some differences could be seen. Reduced OVA-specific IgE levels were seen after RCD405 treatment but not after budesonide treatment. Budesonide and RCD405 reduced the influx of neutrophils, inflammatory macrophages, and alveolar macrophages, whereas a reduction in eosinophils was only seen for budesonide. When comparing RCD405’s effect on type 2 cytokine levels in the lung homogenate and expression of allergy and asthma related genes in lung tissue with that of budesonide, minor differences were seen. Altogether, the small differences in effect between RCD405 and budesonide suggests a similar anti-inflammatory effect of the two compounds, which supports further development of RCD405 as a drug against asthma.

In conclusion, the anti-inflammatory properties of RCD405 may be of interest as an addition to the current treatment armamentarium since its effects are mediated via mechanisms other than those of current medications for asthma and COPD. RCD405 thus has the potential for being a new alternative treatment due to its dual effect with both airway relaxing effects and anti-inflammatory properties. The anti-inflammatory component of RCD405 could also have the potential to reduce the use of ICS and more important, oral corticosteroids. It is possible that one main action of RCD405 is mediated through an inhibition of the NF-κB-pathway and reduced metabolic activity, but further studies are needed to define the mode of action and to investigate whether the anti-inflammatory effects are mediated through similar mechanisms as suggested for the direct-induced relaxation.

### Supplementary Information

**Additional file 1.** Supplemental Methods and Results.

**Additional file 2.** Supplemental Figures.

## Data Availability

Data available within the article or its supplementary materials.

## References

[1] Busse WW (2010). The relationship of airway hyperresponsiveness and airway inflammation: airway hyperresponsiveness in asthma: its measurement and clinical significance. Chest.

[2] Papi A, Brightling C, Pedersen SE, Reddel HK (2018). Pathogenesis of asthma. Lancet.

[3] Peters MC, Wenzel SE (2020). Intersection of biology and therapeutics: type 2 targeted therapeutics for adult asthma. Lancet.

[4] Papi A, Blasi F, Canonica GW, Morandi L, Richeldi L, Rossi A (2020). Treatment strategies for asthma: reshaping the concept of asthma management. Allergy Asthma Clin Immunol.

[5] Hudey SN, Ledford DK, Cardet JC (2020). Mechanisms of non-type 2 asthma. Curr Opin Immunol.

[6] Celli BR, MacNee W, Agusti A, Anzueto A, Berg B, Buist AS (2004). Standards for the diagnosis and treatment of patients with COPD: a summary of the ATS/ERS position paper. Eur Respir J.

[7] Rabe KF, Watz H (2017). Chronic obstructive pulmonary disease. Lancet.

[8] Barnes PJ (2019). Inflammatory endotypes in COPD. Allergy.

[9] Kume H, Hojo M, Hashimoto N. Eosinophil Inflammation and Hyperresponsiveness in the Airways as Phenotypes of COPD, and Usefulness of Inhaled Glucocorticosteroids. Front Pharmacol. 2019;10. Available from: 10.3389/fphar.2019.00765. PMC6676333 31404293

[10] Soler-Cataluña J, Martínez-García MÁ, Sánchez PR, Salcedo E, Navarro M, Ochando R (2005). Severe acute exacerbations and mortality in patients with chronic obstructive pulmonary disease. Thorax.

[11] Mukherjee S, Pal M (2013). Quinolines: a new hope against inflammation. Drug Discov Today..

[12] da Silva EBS, da Silva MAJ, de Sousa NRT, da Rocha PI, da Rocha PGM (2022). New trends in biological activities and clinical studies of quinolinic analogues: a review. Curr Drug Targets..

[13] Tanner L, Single AB (2019). Animal models reflecting chronic obstructive pulmonary disease and related respiratory disorders: translating pre-clinical data into clinical relevance. J Innate Immun.

[14] Lee SY, Cho JH, Cho SS, Bae CS, Kim GY, Park DH (2018). Establishment of a chronic obstructive pulmonary disease mouse model based on the elapsed time after LPS intranasal instillation. LAR.

[15] Zhang Q, Lenardo MJ, Baltimore D (2017). 30 years of NF-κB: a blossoming of relevance to human pathobiology. Cell.

[16] Naline E, Zhang Y, Qian Y, Mairon N, Anderson GP, Grandordy B (1994). Relaxant effects and durations of action of formoterol and salmeterol on the isolated human bronchus. Eur Respir J.

[17] Adner M, Larsson B, Säfholm J, Naya I, Miller-Larsson A (2010). Budesonide prevents cytokine-induced decrease of the relaxant responses to formoterol and terbutaline, but not to salmeterol, in mouse trachea. J Pharmacol Exp Ther..

[18] Skogvall S, Dalence-Guzmán MF, Berglund M, Svensson K, Mesic A, Jönsson P (2008). Discovery of a potent and long-acting bronchorelaxing capsazepinoid, RESPIR 4–95. Pulm Pharmacol Ther..

[19] Larsson OJ, Manson ML, Starkhammar M, Fuchs B, Adner M, KumlienGeorén S (2016). The TLR7 agonist imiquimod induces bronchodilation via a nonneuronal TLR7-independent mechanism: a possible role for quinoline in airway dilation. Am J Physiol Lung Cell Mol Physiol..

[20] Deshpande DA, Wang WCH, McIlmoyle EL, Robinett KS, Schillinger RM, An SS (2010). Bitter taste receptors on airway smooth muscle bronchodilate by localized calcium signaling and reverse obstruction. Nat Med.

[21] Tanner L, Bergwik J, Bhongir RK V, Pan L, Dong C, Wallner O, et al. Pharmacological OGG1 inhibition decreases murine allergic airway inflammation. Front Pharmacol. 2022;13. Available from: 10.3389/fphar.2022.999180. PMC9619105 36324676

[22] Sharma P, Yi R, Nayak AP, Wang N, Tang F, Knight MJ (2017). Bitter taste receptor agonists mitigate features of allergic asthma in mice. Sci Rep..

[23] Nials AT, Uddin S (2008). Mouse models of allergic asthma: acute and chronic allergen challenge. Dis Model Mech.

[24] McKenzie GJ, Bancroft A, Grencis RK, McKenzie ANJ (1998). A distinct role for interleukin-13 in Th2-cell-mediated immune responses. Curr Biol.

[25] Nakanishi A, Morita S, Iwashita H, Sagiya Y, Ashida Y, Shirafuji H (2001). Role of gob-5 in mucus overproduction and airway hyperresponsiveness in asthma. Proc Natl Acad Sci U S A..

[26] Xu W, Ghosh S, Comhair SAA, Asosingh K, Janocha AJ, Mavrakis DA (2016). Increased mitochondrial arginine metabolism supports bioenergetics in asthma. J Clin Invest.

[27] Asosingh K, Lauruschkat CD, Alemagno M, Frimel M, Wanner N, Weiss K (2020). Arginine metabolic control of airway inflammation. JCI Insight..

[28] Niese KA, Collier AR, Hajek AR, Cederbaum SD, O’Brien WE, Wills-Karp M (2009). Bone marrow cell derived arginase I is the major source of allergen-induced lung arginase but is not required for airway hyperresponsiveness, remodeling and lung inflammatory responses in mice. BMC Immunol.

[29] Barron L, Smith AM, El Kasmi KC, Qualls JE, Huang X, Cheever A (2013). Role of arginase 1 from myeloid cells in th2-dominated lung inflammation. PLoS ONE.

[30] Cloots RHE, Sankaranarayanan S, Poynter ME, Terwindt E, van Dijk P, Lamers WH (2017). Arginase 1 deletion in myeloid cells affects the inflammatory response in allergic asthma, but not lung mechanics, in female mice. BMC Pulm Med.

[31] Boonpiyathad T, Satitsuksanoa P, Akdis M, Akdis CA. Il-10 producing T and B cells in allergy. Semin Immunol. 2019;44:101326. 10.1016/j.smim.2019.101326 31711770

[32] Nassef MZ, Hanke JE, Hiller K (2021). Mitochondrial metabolism in macrophages. Am J Physiol Cell Physiol.

[33] Pan S, Conaway S, Deshpande DA (2019). Mitochondrial regulation of airway smooth muscle functions in health and pulmonary diseases. Arch Biochem Biophys.

[34] Shang XF, Morris-Natschke SL, Liu YQ, Li XH, Zhang JY, Lee KH (2022). Biology of quinoline and quinazoline alkaloids. Alkaloids Chem Biol.

[35] Jin HZ, Lee JH, Lee D, Lee HS, Hong YS, Kim YH (2004). Quinolone alkaloids with inhibitory activity against nuclear factor of activated T cells from the fruits of Evodia rutaecarpa. Biol Pharm Bull.

[36] Yoon JS, Jeong EJ, Yang H, Kim SH, Sung SH, Kim YC (2012). Inhibitory alkaloids from Dictamnus dasycarpus root barks on lipopolysaccharide-induced nitric oxide production in BV2 cells. J Enzyme Inhib Med Chem.

[37] Ratheesh M, Sindhu G, Helen A (2013). Anti-inflammatory effect of quinoline alkaloid skimmianine isolated from Ruta graveolens L. Inflamm Res.

