# Supplementary material for: A novel quinoline with airway relaxant effects and anti-inflammatory properties
Source: Respir Res. 2024 Mar 30;25:146. doi: 10.1186/s12931-024-02780-8 (PMC10981829; doi:10.1186/s12931-024-02780-8)
Supplement: Supplementary file 1 — Additional file 1. Supplemental Methods and Results. [file 12931_2024_2780_MOESM1_ESM.docx]

**SUPPLEMENTAL METHODS AND RESULTS**

**Receptor binding assay**

The *in vitro* binding of RCD405 to β2-adrenergic, M1, M2 and M3 recombinant receptors (expressed in CHO-cells) was compared to radiolabelled reference ligands to each of the different receptors. The reference ligands used were alprenolol for the adrenergic receptors and atropine for the muscarinic receptors (1-3).

**Assay to measure effects on isolated mitochondrial OXPHOS complexes**

The MitoTox™ Complete OXPHOS Activity Assay Kit (Abcam, Cambridge, UK) was used to study the effects of RCD405 on mitochondrial OXPHOS complexes as per the instructions provided by the manufacturer.

**SUPPLEMENTAL RESULTS**

**Binding assay**

The total binding at 100 µM of RCD405 was 19.0% for the β2-adrenergic receptor and 11.1, 3.2 and -5.7% (mean values, n=2) for the M1, M2 and M3 receptors, respectively (Supplementary figure 10). All values below 25% were considered not significant and within normal variability.

**Effects of RCD405 on isolated mitochondrial OXPHOS complexes**

Effects from RCD405 on mitochondrial OXPHOS complexes I, II, IV and V (Supplementary figure 11). Data for complex II&III are not shown since the progression of the assay was too fast and the substrate was consumed before the reaction ended. No inhibition of complex I by RCD405 was observed. Since the progression of the assay for complex III was very fast, the substrate was most likely completely consumed before the reaction ended, and the results should therefore be interpreted with caution.

**References**

1. Joseph SS, Lynham JA, Colledge WH, Kaumann AJ. Binding of (-)-[3H]-CGP12177 at two sites in recombinant human beta 1-adrenoceptors and interaction with beta-blockers. Naunyn Schmiedebergs Arch Pharmacol. 2004;369:525-32.

2. Dörje F, Wess J, Lambrecht G, Tacke R, Mutschler E, Brann MR. Antagonist binding profiles of five cloned human muscarinic receptor subtypes. J Pharmacol Exp Ther. 1991;256:727-33.

3. Peralta EG, Ashkenazi A, Winslow JW, Smith DH, Ramachandran J, Capon DJ. Distinct primary structures, ligand-binding properties and tissue-specific expression of four human muscarinic acetylcholine receptors. EMBO J. 1987;6:3923-9.
